# Modelling of Nonthermal Dielectric Barrier Discharge Plasma at Atmospheric Pressure and Role of Produced Reactive Species in Surface Polymer Microbial Purification

**DOI:** 10.3390/polym15051235

**Published:** 2023-02-28

**Authors:** Samira Elaissi, Norah A. M. Alsaif

**Affiliations:** Department of Physics, College of Science, Princess Nourah Bint Abdulrahman University, P.O. Box 84428, Riyadh 11671, Saudi Arabia

**Keywords:** nonthermal atmospheric plasma, dielectric barrier discharge, polymer surface, decontamination, atmospheric pressure, modelling

## Abstract

A nonthermal atmospheric plasma reactor was used to sterilize polymer surfaces and satisfy safety constraints in a biological medium. A 1D fluid model was developed using COMSOL Multiphysics software^®^ 5.4 with a helium–oxygen mixture at low temperature for the decontamination of bacteria on polymer surfaces. An analysis of the evolution of the homogeneous dielectric barrier discharge (DBD) was carried out through studying the dynamic behavior of the discharge parameters including the discharge current, the consumed power, the gas gap voltage, and transport charges. In addition, the electrical characteristics of a homogeneous DBD under different operating conditions were studied. The results shown that increasing voltage or frequency caused higher ionization levels and maximum increase of metastable species’ density and expanded the sterilization area. On the other hand, it was possible to operate plasma discharges at a low voltage and a high density of plasma using higher values of the secondary emission coefficient or permittivity of the dielectric barrier materials. When the discharge gas pressure increased, the current discharges declined, which indicated a lower sterilization efficiency under high pressure. A short gap width and the admixture of oxygen were needed for sufficient bio-decontamination. Plasma-based pollutant degradation devices could therefore benefit from these results.

## 1. Introduction

Bio-decontamination technologies have emerged due to the development of compatible polymers used in biomedical applications such as medical implants. These heat-sensitive materials require both sterile handling and substrate activation before use. Thus, several pre-treatments are needed to obtain the desirable physicochemical surface properties that satisfy safety constraints in the biological medium [1,2,3].

Sterilization methods using an autoclave or an oven, ethylene oxide, gamma irradiation, and ultraviolet radiation are commonly applicable [4,5,6]. However, most of these processes operate in closed spaces, require expensive and complex equipment, and need a lengthy sterilization [7], while others are toxic to human body and difficult to apply to air purifiers as germicidal agents [8]. In addition, traces of active compounds often remain after the application of these techniques and generate an unacceptable risk [9]. Consequently, these methods constitute an inadequate solution for microbial decontamination at ambient temperature. 

Nowadays, a particular interest has developed in non-thermal plasma as a useful method to kill bacteria or inhibit their growth [10,11,12]. The moderate neutral gas temperatures and the benefits associated with gas phase processes [13], enable plasma to modify and bio-sterilize small and complex thermolabile polymeric surfaces. Various conventional sterilization methods including heat (steam and dry heat), chemical agents (ethylene oxide), and radiation (ultraviolet and gamma irradiation) are not suitable for heat-sensitive polymer sterilization [14]. The advantages of heat treatment include its effectiveness, speed, simplicity, and the lack of toxic residues. However, it is difficult to sterilize biodegradable polymers using heat treatment, since they have a low glass transition temperature. Furthermore, steam sterilization can cause the hydrolytic degradation of the material to be sterilized due to the existence of vaporized water [15]. On the other hand, despite its rapidity simplicity and effectiveness, radiation sterilization alters scaffold materials’ chemical properties, reduces their compressive mechanical characteristics, and increases their degradation rates after sterilization. In addition, ultraviolet disinfection has limitations, including the lack of a lasting residual disinfection ability, and dark repair and photoreactivation may occur [16]. Specifically, atmospheric plasma offers a great advantage, since it can be developed in the open air with a small energy consumption and without the need of the expensive installation of a vacuum system.

Among its properties, plasma can generate a mixture of reactive oxygen and nitrogen species (RONS) including charged particles, radicals, and metastable molecules, as well as an electric field and UV radiation. These latter are considered effective sterilizing agents capable to inactivate microorganisms such as *Bacillus atrophaeus*, whose spores may lie on synthetic and natural polymer products such as polyethylene and polystyrene polymers, which are commonly used because of their relevance in biomedical applications [17]. *B. atrophaeus* may present in an inactive or dormant form that is resistive to chemical and physical agents like radiation, heat, changes in pH, and various chemicals compared to the actively growing vegetative form [18].

As a result, non-thermal atmospheric gas discharge plasma processes are the recommended technique in this case. Indeed, they induce physical and chemical changes of the external layer of the polymers’ surface, and no change produced to their bulk properties and simultaneously allowing the sterilization of the surface in a short time in an efficient and economically viable way [19].

However, in addition to inactivating microorganisms, plasma-generated species can also react with polymer surfaces, causing changes in wettability, roughness, and elemental composition which can alter their functionalities [20,21]. Therefore, the plasma mechanism of antimicrobial treatment needs to be examined in detail.

A variety of configurations have been reported for electrical discharge deviation [22,23]. Among the most working setups used is the DBD for a cold atmospheric pressure electric discharge [24]. In this system, a dielectric layer covers at least one electrode of the DBD arrangement and may consist of glass, quartz, or ceramics. The DBD cell can be powered by a repetitive pulsed power or an alternating voltage, limiting the discharge current and preventing arc discharges. As a result of its uncomplicated configuration and removing of a vacuum system, industrial gas dis-charge applications are also well suited to the DBD [25]. Nonetheless, the small electrode gap in discharge geometries is generally lower than a few millimeters, which makes the understanding discharge physics challenging [26]. Therefore, numerical modelling is required in plasma discharge diagnostics to anticipate the interactions between charged particles in the discharge. 

Among the numerical simulations of atmospheric pressure plasmas extensively applied in this field is the 2D fluid model used by Gadkari et al. [27]. An investigation of the partial packing impact on the coaxial DBD reactor characteristics in helium plasma was conducted. In the study by Pan et al., the DBD spatiotemporal characteristics at 1 atm were stimulated numerically by using the fluid model [28]. Kanazawa et al. showed that a homogeneous and stable barrier discharge at atmospheric pressure requires some specific conditions, such as using helium or argon gases in various mixtures and a high-frequency oscillating source exceeding 1 kHz. Golubovskii et al. investigated the effect of elementary reactions on the homogeneous DBD helium discharge and investigated its spatiotemporal characteristics using a 1-D fluid model [29]. At atmospheric pressure with a few millimeters gap, several electrical, acoustic, and infrared measurements showed that a controlled homogeneous barrier discharge could be obtained ((p × d)~500 torr cm) [30], although it was unclear if this discharge behaved like a glow discharge at low pressure or if it was caused by multiple filaments coupled together.

In this research study, a DBD numerical model in helium–oxygen plasma at atmospheric pressure was developed using COMSOL Multiphysics software^®^ 5.4 [31]. Our objective was to study the spatial and temporal evolution of the different parameters of discharge and to numerically examine the density distribution of reactive species to show their effect on the microbial inactivation rate. Based on the optimization of external parameters including external voltage frequency, dielectric thickness, interelectrode width, and amplitude, the discharge parametric behavior was analyzed. The main purpose of this paper was to develop an efficient system of sterilization that utilizes a pulsed DBD and is characterized by its appropriate germicidal effect.

## 2. Microbial Inactivation Setup

**Figure 1:**
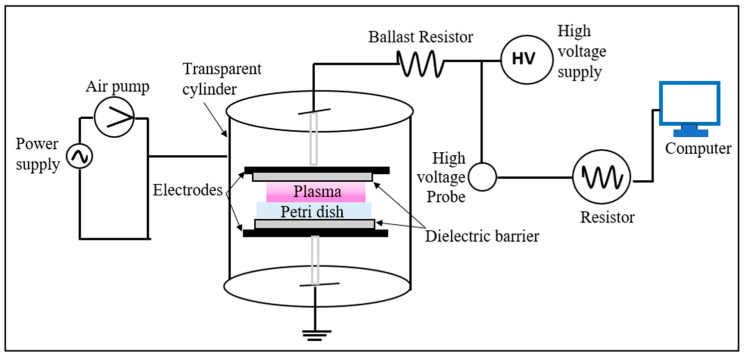
Experimental setup. Schematic diagram of the DBD reactor.

Figure 1 describes the experimental microbial inactivation setup [32]. In this system, a sinusoidal alternating high-voltage power supply drives the plasma with pulse frequency and voltage equal to 27 kHz and 18 kV, respectively. The pulse rise time is 3 ms. The temperature of the gas is 300 K, and the pressure is 1.01 × 10^5^ Pa. The discharge gas is a helium–oxygen mixture (1% O_2_), and the plasma treatment duration is 1 min.

**Figure 2:**
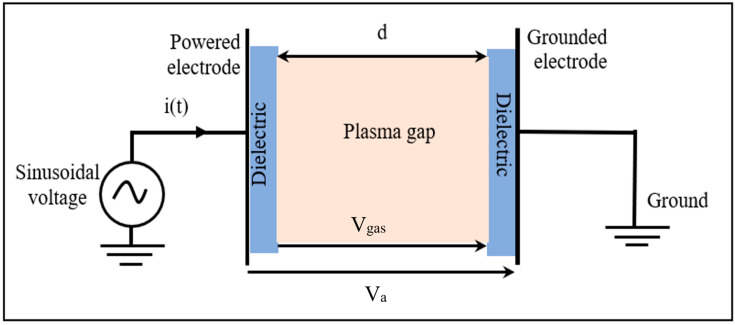
Schematic diagram of the DBD plasma gap.

A DBD was generated between the two planar copper electrodes at a 0.2 cm distance (Figure 2). Round electrodes with a 5 cm radius and 0.2 mm thickness were used, which were covered with alumina dielectric layers (ε_r_ = 10) of 0.1 cm in thickness [33].

## 3. Model Description

### 3.1. Numerical Model Equations

A self-consistent fluid model describes the DBD cold plasma using balance equations derived from Boltzmann transport equations [34]. If no radial effects influence the main characteristics of the plasma, a one-dimensional discharge description is performed considering only the axial component x [35].

For all the considered species, continuity and momentum equations are solved using the drift-diffusion flux approximation [36,37,38]. The system of equations used is resolved by coupling the transport equations and the Poisson equation to obtain the electric field. The discharge is governed by the following equations:

Continuity equation:

(1) $$\frac{\partial n_{i}}{\partial t}+\nabla \cdot \Gamma_{i}=S_{i}$$

where $n_{i}$ represents the density of electrons (e), ions (p), and excited particles (m), and $\Gamma_{i}$ represents the flux density, according to [39]:

(2) $$\Gamma_{i}=-\mu_{i}n_{i}E-\frac{\partial }{\partial x}(D_{i}n_{i})$$

where E represents the electric field, and $\mu_{i}$ and $D_{i}$ represent the mobilities and the diffusion coefficients of each type of particle. $S_{i}$ represents the source terms for electrons according to [40]:

(3) $$S_{i}=\sum_{j=1}^{M}x_{j}\alpha_{j}N_{n}\left|\Gamma_{e}\right|$$

where M is the number of reactions, $x_{j}$ and  $\alpha_{j}$ denote the molar fraction of the target species and the Townsend coefficient for the jth reaction respectively, and $N_{n}$ represents the total number of neutral particles.

The Equation of electron energy balance:

(4) $$\frac{\partial n_{\epsilon }}{\partial t}+\nabla \cdot \Gamma_{\epsilon }=S_{\epsilon }$$

(5) $$\Gamma_{\epsilon }=-\frac{5}{3}\mu_{e}n_{e}\epsilon E-\frac{5}{3}D_{e}\nabla \cdot n_{e}\epsilon$$

Note that ϵ denotes the mean electron energy. $S_{\epsilon }$ represents the source terms for the energy equation as follows:

(6) $$S_{\epsilon }=\sum_{j=1}^{P}x_{j}\alpha_{j}N_{n}\left|\Gamma_{e}\right|\Delta \epsilon_{j}$$

P is the number of non-elastic collisions of an electron, and $\Delta \epsilon_{j}$ is the energy dissipation from the jth reaction.

For the electric field computation, the Poisson equation is coupled with the balance equations [41]:

(7) $$\frac{\partial^{2}V}{\partial x^{2}}=-\frac{q}{\varepsilon }\left(n_{p}-n_{e}\right)$$

where q denotes the unit charge, and ε is the dielectric permittivity. The electric field is resolved inside the dielectrics and in the plasma region and defined as the negative gradient of the electric potential:

(8) $$E=-\frac{\partial V}{\partial x}$$

The electrical properties of the discharge, including the applied voltage $V_{\mathrm{app}}\left(t\right)$, the gas voltage $V_{g}\left(t\right),$ and the discharge current $I_{d}$, are expressed by [42]:

(9) $$V_{\mathrm{app}}\left(t\right)=V_{m}\sin\left(2\pi f\times t\right)$$

(10) $$V_{g}\left(t\right)=V_{\mathrm{app}}\left(t\right)-V_{\mathrm{sd}}\left(t\right)=V_{\mathrm{app}}\left(t\right)-\frac{1}{C_{\mathrm{sd}}}\int_{t_{0}}^{t}I_{d}\mathrm{dt}$$

(11) $$I_{d}\left(x,t\right)=\left|q\right|\cdot S\int_{t_{0}}^{t}\int_{0}^{d}\left|\Gamma_{e}-\Gamma_{p}\right|\mathrm{dx}\;\mathrm{dt}$$

where $V_{m}$ and f represent the amplitude and the frequency of the applied voltage, respectively. $V_{\mathrm{sd}}\left(t\right)$ represents the solid dielectrics voltage, $C_{\mathrm{sd}}$ its capacitance, and S is the area of the electrode.

### 3.2. Boundary and Initial Conditions

The model considers the effect of the dielectric covering the electrodes, since the discharge is a DBD. Gauss’s law is used to describe the influence of charge accumulation on the dielectric metallic at the interface between the dielectric and the plasma [43]:

(12) $$\varepsilon_{\mathrm{diel}}E_{\mathrm{diel}}\cdot u_{n}-\varepsilon_{\mathrm{gas}}E_{\mathrm{gas}}\cdot u_{n}=\sigma$$

E_gas_ and E_diel_ represent, respectively, the electric field in the gas discharge and inside the dielectric; $\varepsilon_{\mathrm{diel}}$ and $\varepsilon_{\mathrm{gas}}$ are the permittivity of the dielectric surface and of the gas, respectively; $u_{n}$ represents the unit vector pointing normally to the wall, where the charge accumulation takes place. The surface charge density on the dielectric is σ and is calculated by dividing the charge particle flux directed to the surface at the cathode and anode [44]:

(13) $$\frac{\partial \sigma {\left(t\right)}_{\mathrm{Cath}}}{\partial t}=\left|q\right|(\Gamma_{p}\left(1+\gamma_{\sec}\right))$$

(14) $$\frac{\partial \sigma {\left(t\right)}_{\mathrm{Anod}}}{\partial t}=\left|q\right|\left(\Gamma_{p}-\Gamma_{e}\right)$$

where $\gamma_{\sec}$ is the secondary electron emission term.

The electric potential is V = $V_{\mathrm{app}}$ at the powered electrode and V = 0 at the ground electrode. The flux particles and the space density boundary values are as follows: 

at the cathode, $\Gamma_{e}=-\gamma_{\sec}{\;\Gamma }_{p}$ and $\nabla n_{p}=\nabla n_{m}=0$

at the anode, $\nabla n_{p}=\nabla n_{e}=\nabla n_{m}=0$

The initial conditions consist of uniformly distributed electrons, ions, metastable state, and surface charge: $n_{e}$ (t = 0) = $n_{p}$ (t = 0) = 10^16^ cm^−3^, n_m_ (t = 0) = 10^9^ cm^−3^, and σ (t = 0) = 10^−8^ C/cm^2^.

The chemistry model included in our simulation considers the chemical reactions and the production and loss rates of different species. The reactions of pure helium and of the helium–oxygen mixture are shown in Refs. [45,46].

### 3.3. Computational Study

For modelling the plasma behavior and plasma properties of homogeneous DBD discharge, the COMSOL Multiphysics^®^ 5.4 time-dependent module was used [47]. A 1D fluid model of the helium–oxygen mixture was applied to the parallel-plate geometry at atmospheric pressure while assuming the local electric field approximation. An efficient finite element method led to a reasonable resolution of these partial differential equations. Since particle transport equations and Poisson’s equations are strongly coupled, it was imperative to adopt a very appropriate computation time step to obtain a rapid computational time evolution for the physical phenomena’s convergence. The numerical simulation was performed using a backward differentiation formula (BDF) solver algorithm [48].

## 4. Results

The electrical representation of the voltage and the discharge current are illustrated in one cycle. In addition, the variations of the important physical quantities resulting from the numerical modelling were investigated, first for helium discharge, to validate the simulation model and in a second time, for the helium–oxygen mixture (He-1% O_2_), to study the inactivation of *Bacillus atrophaeus* spores on polymer surfaces. Moreover, the efficiency of inactivation was examined according to the process gas and the distance of operation.

### 4.1. Helium DBD Discharge

The helium DBD plasma discharge was induced by 1300 V sinusoidal alternating-voltage power supply at 10 kHz. The gap distance was 0.5 mm.

#### 4.1.1. Discharge Structure

**Figure 3:**
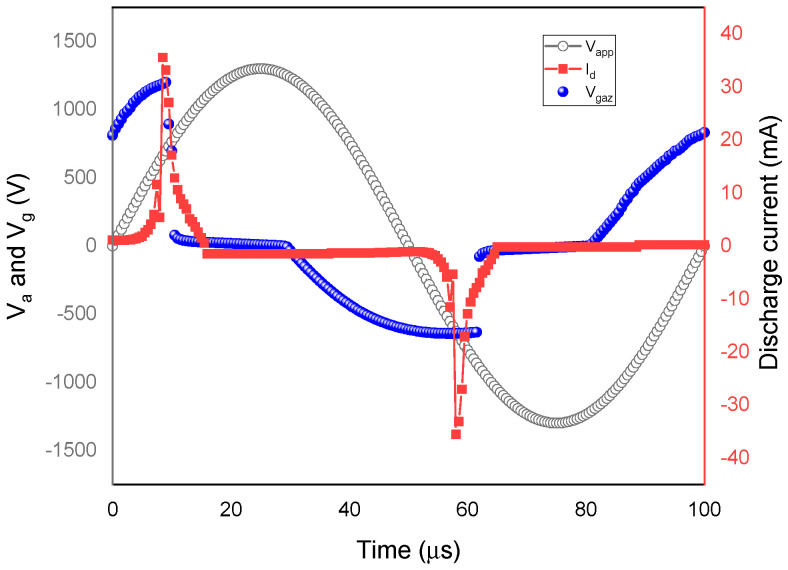
Evolution during one cycle of the calculated applied voltage, gas voltage, and discharge current of the DBD.

The evolution of the discharge current $I_{d}\left(t\right)$, the implemented voltages $V_{\mathrm{app}}\left(t\right)$, and the gas voltage $V_{g}\left(t\right)$ during one cycle is plotted in Figure 3. First, a sudden increase in current occurred during the positive half-voltage discharge from 1 mA to a maximum of 32 mA. A breakdown of the gas occurred with the abruptly change in current, and the discharge lasted about 5 µs. Simultaneously, the gas voltage $V_{g}$ changed from 760 V at t = 0 s to 1200 V at t = 9 µs, at which voltage, the first discharge appeared. As the current peak was reached, $V_{g}$ caused the extinction of the discharge, and a negative voltage $V_{g}$ was triggered, announcing the ignition of the second discharge through the subsequent half cycle.

The discharge current maintained its behavior with a negative $V_{\mathrm{app}}$, but in the opposite direction, and the negative peak reached 34 mA. According to the discharge current behavior, the DBD exhibited one breakdown each half-applied voltage cycle in the atmospheric glow regime. Hence, there were two breakdown events in each cycle. Throughout the discharge interval, the gas voltage and the discharge current profile followed the external voltage periodicity. At each half cycle, an opposite voltage called dielectric voltage, $V_{\mathrm{sd}}$, was generated by accumulating charges within the dielectric barrier’s inner layers. Finally, the discharge was suppressed when the voltage $V_{g}$ decreased, which prevented the electric arc generation and the formation of cold plasma.

#### 4.1.2. Predicted Electric Field and Species Distribution

**Figure 4:**
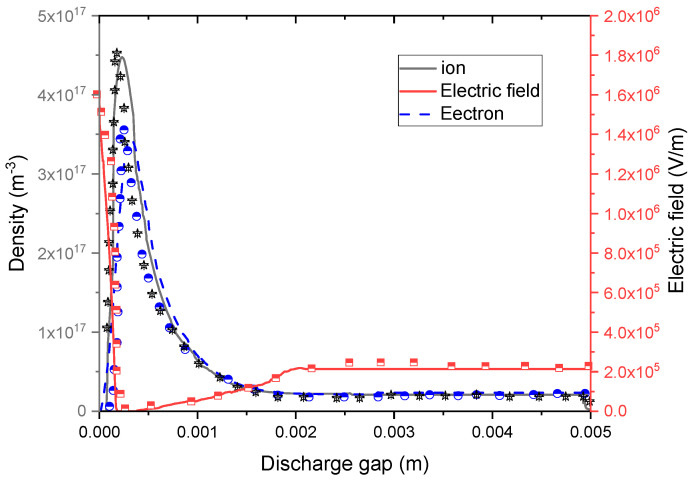
Comparison of calculated and experimental data [50]. Spatial distribution of the electron and ion densities and the electric field in helium plasma at a maximum discharge current time. The right side indicates the anode, and the left side indicates the cathode.

The spatial distribution of the charged particle densities and of the electric field are displayed in Figure 4 at the maximal discharge current (t = 10 µs). The discharge was characterized by four discharge regions like those of DC glow discharge at low pressure [49]. We found (i) a high cathode-fall region, that exhibited a maximal electric field of 16.5 kV/cm resulting from the wide positive space charges close to the cathode.

In this zone, limited to 0.3 mm, the ion density reached a maximum of 4.7 × 10^17^ m^−3^. The electron density attained a maximum of 3.6 × 10^17^ m^−3^; (ii) a second negative-glow region extending up to 0.73 mm in length, in which the densities of electrons and ions were equal, and the electric field remained small; (iii) the faraday dark space, with 1.4 mm thickness, where the ion and electron densities were in close proximity to each other as the electric field increased. Here, a little negative space charge occurred; (iv) a positive-column zone, occupying the greatest area, whose width was 2.58 mm. In this electrically neutral plasma region, the ion and electron densities were equal and close to 2 × 10^16^ m^−3^. The electric field was relatively low, corresponding to 2 kV/cm. The electron mobility was reduced by the interaction with the ions.

**Figure 5:**
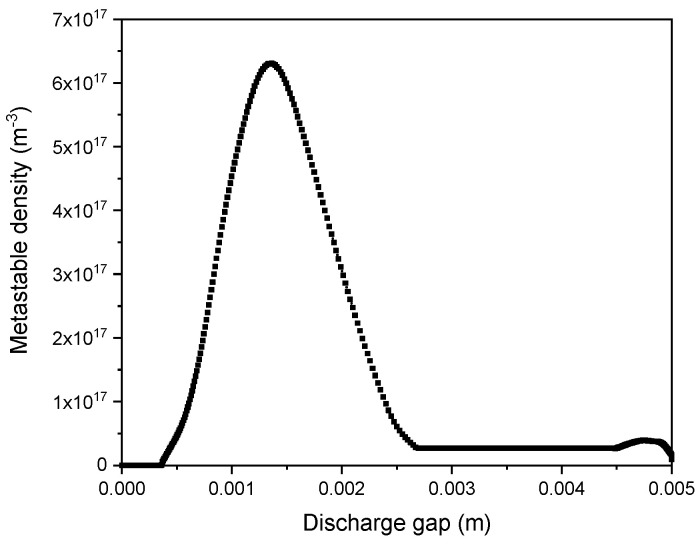
Metastable density (He*) spatial distribution at maximum discharge current.

Figure 5 shows the density spatial distribution of metastable helium when there was a maximum discharge current. The metastable density distribution showed a profile similar to the ion and electron density profiles. During the cathode-fall region, a maximum density of 6.3 × 10^17^ m^−3^ was reached, while in the positive-column region, it was constant, with a value of 2 × 10^16^ m^−3^.

For validation, it was found that the numerical simulation results of the discharge parameters matched the literature results developed in the same context [50].

### 4.2. Helium–Oxygen DBD Discharge

The helium–oxygen plasma discharge, He/O_2_ (1% O_2_), was operated by a 30 kV high-voltage sinusoidal alternating power supply at 10 kHz. The gap distance was 0.2 mm [51].

**Figure 6:**
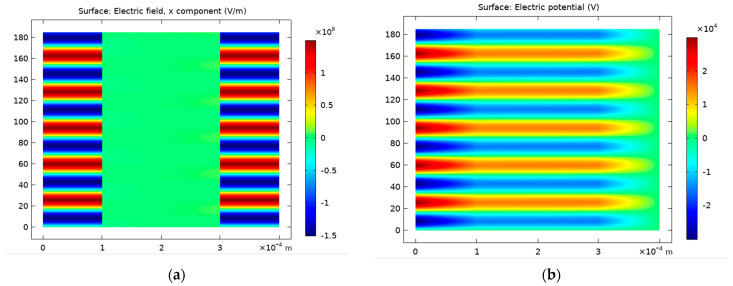
Spatiotemporal evolution of the electric field (**a**) and potential (**b**) in the interelectrode gap.

Figure 6 illustrates the spatiotemporal evolution of the electric field (Figure 6a) and potential (Figure 6b) in the interelectrode distance. Indeed, the potential and the electric field changed as a function of the position. The length of the sheath region could be determined with the electric field root-mean-square value [52]. Due to the plasma diamagnetic property, the electric field in the inter-electrode distance was reduced when there was an electrical discharge.

**Figure 7:**
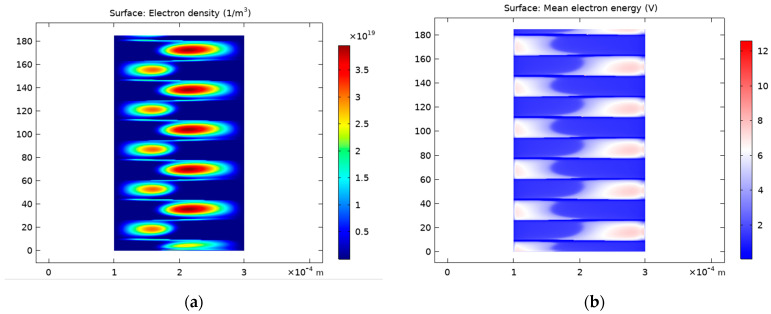
Spatiotemporal distribution of electron density (**a**) and temperature (**b**) versus the gap width.

Figure 7a illustrates the spatiotemporal distribution of electron density versus the gap extension. Indeed, two discharges occurred in each cycle, one in the positive half of the voltage cycle, and the other in the negative one. The first discharge event occurred close to the powered electrode, on the left-hand side of the picture. The maximum electron density reached approximately 3.5 × 10^19^ m^−3^. Figure 7b shows the electron temperature evolution at the gap center. Due to the two discharges events in a cycle, as shown in Figure 3, the electron temperature changed twice in each period.

### 4.3. Role of Different Radical Species Produced by a Dielectric Barrier Discharge in Microbial Inactivation

In a DBD discharge, a large amount of charged particles collides with N_2_, O_2_, and H_2_O, generating active particles and free radicals along with ultraviolet radiation and shock waves. Both oxygen and nitrogen reactive species (RONS) are generated in atmospheric plasma through complex chemistry [53]. Experiments from the literature, including photographs taken with a Vis–IR digital camera confirmed the formation of atomic oxygen, and light emission spectra affirmed the generation of oxygen radicals during plasma operation [54]. These reactive radicals play a significant role in (i) killing bacteria, (ii) causing membrane damage, and (iii) degrading DNA [55].

This study excluded the influence of plasma heat on inactivating *B. atrophaeus* spores. Indeed, a variety of proteins in these bacteria increase their resistance to chemical and physical antimicrobial agents [56]. Further, atmospheric pressure plasma operates in a burst mode which reduces the temperature on the target, while maintaining antimicrobial effectiveness [57].

Oxygen admixed with helium plasma generates reactive species such as atomic oxygen, oxygen molecules in excited state, and ozone, that have antibacterial properties, which are responsible for increasing bacterial inactivation significantly. Hence, plasma disinfection and surface processing are primarily driven by reactions initiated by reactive oxygen and nitrogen reactive species (RONS) [58].

**Figure 8:**
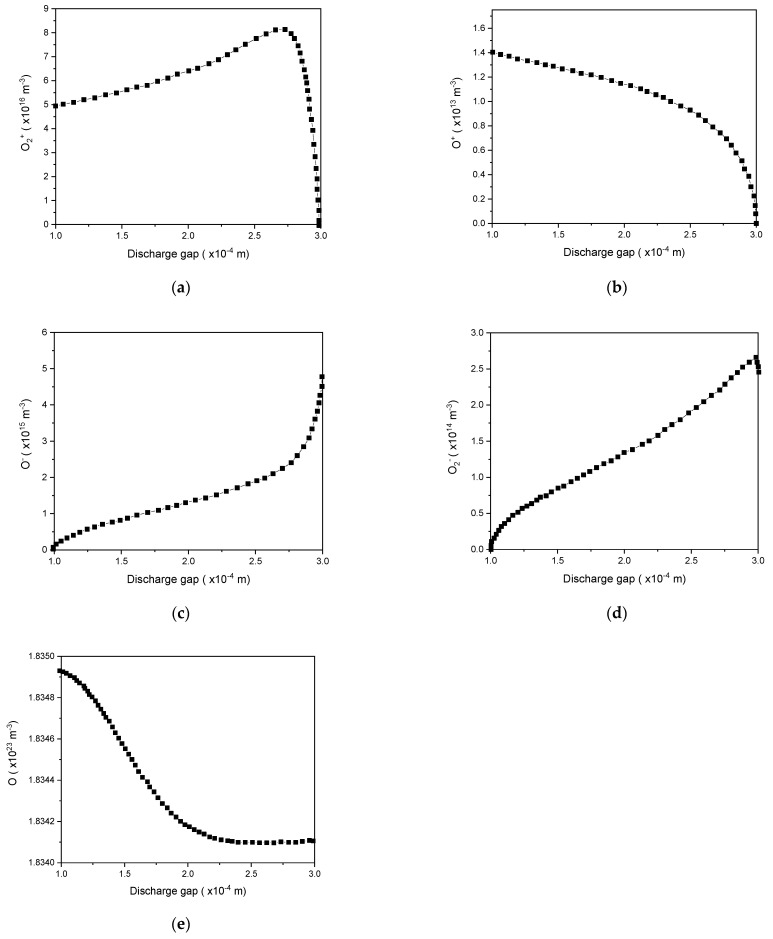
Density distribution of O_2_^+^ (**a**), O^+^ (**b**), O_2_ (**c**), O^−^ (**d**), and O (**e**) in the gap at the maximum discharge current.

As illustrated in Figure 8, the density distribution of different oxygen species obtained from the numerical model indicated that the density of O_2_^+^, O^−^, and O was considerable among those of all reactive oxygen species considered (O_2_^+^, O^+^, O_2_^−^, O^−^, and O). Subsequently, plasma disinfection and surface processing were primarily determined by the initiated reactions of atomic oxygen and excited oxygen molecules [59]. This confirmed the results of Dobrynin et al. [60] who illustrated that oxygen is essential for a speedy as well as efficient sterilization process, regardless of the composition of the used gas.

**Figure 9:**
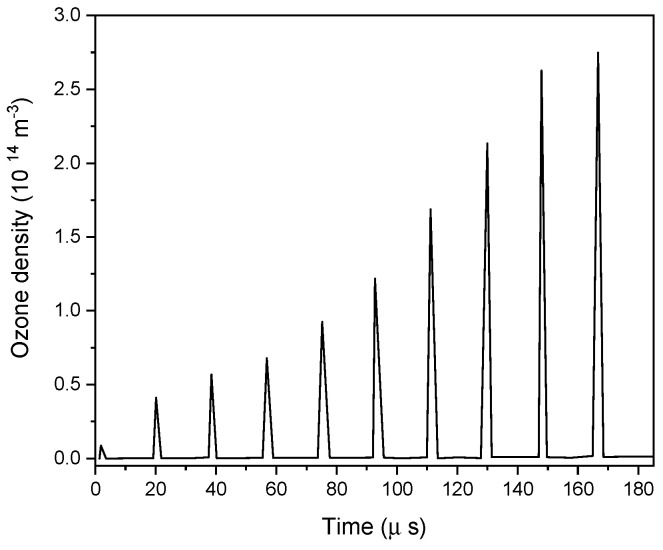
Ozone production and accumulation in the DBD reactor as a function of time.

Figure 9 illustrates the significant growing density of ozone. The results showed that ozone accumulated in the gap during every discharge event, reaching a density of 2.8 × 10^14^ m^−3^.

### 4.4. Power Distribution

With the proposed model, it was possible to determine the average consumed power by the DBD as follows [61]:

(15) $$P=\frac{1}{T}\int_{0}^{T}V_{g}\left(t\right)I_{d}\left(t\right)\mathrm{dt}$$

**Figure 10:**
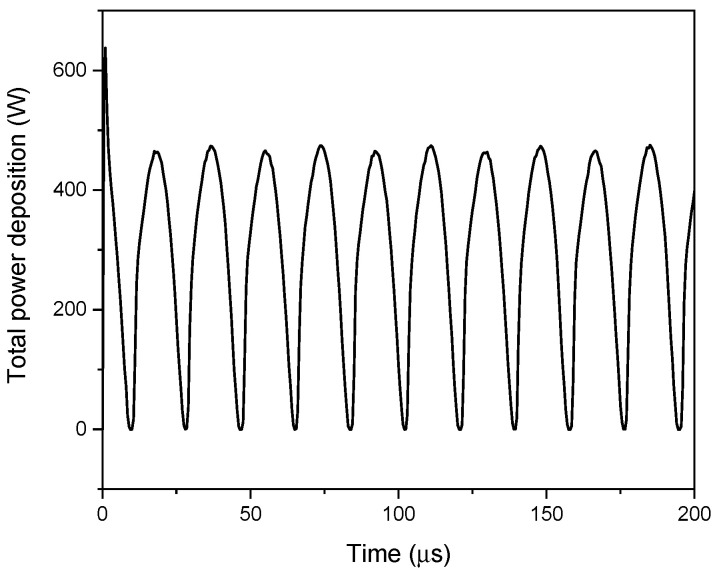
Time evolution of the total power distribution at x = 0.2 mm (the center of the gap).

In Figure 10, the total power distribution in the center of the gap is shown and appears to change two times in one period, due to two discharges phenomena per period [62]. According to Equation (15), the power rose with the voltage, indicating an increase in efficiency.

### 4.5. Influence of the Operational Conditions

#### 4.5.1. Influence of External Voltage and Frequency Modulation

**Figure 11:**
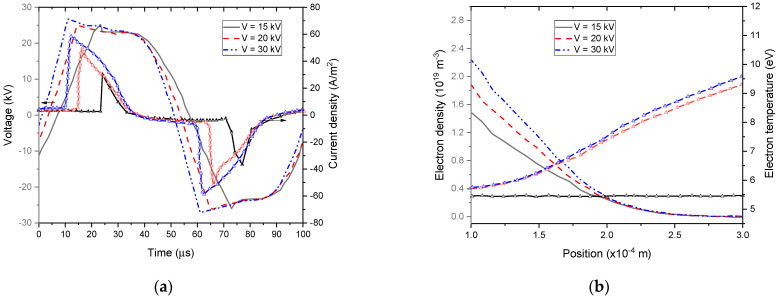
Calculated current density and gas voltage waveforms (**a**) and spatial electron density and temperature distribution at maximum discharge current (**b**) for different external voltages.

**Figure 12:**
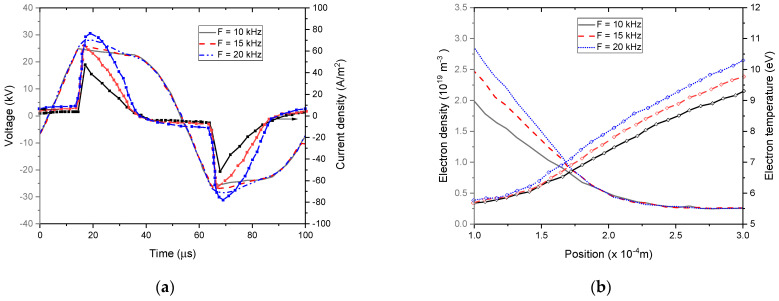
Spatiotemporal distribution of the electron density (**a**) and temperature (**b**) versus the gap extension.

Figure 11 and Figure 12 represent the variations of current, gas voltage, electron density, and temperature depending on external voltage amplitude and frequency for the He–O_2_ (1% O_2_) plasma gas.

Particularly, Figure 11a and Figure 12a show a proportional relationship between the voltage gas amplitude on the one side and the current density on the other for different amplitude and frequencies of the external voltage. It was observed that the shape of the gas voltage profiles remained the same, whereas the frequency and amplitude of the external voltage had a significant impact on the current density profiles [63].

Figure 11b and Figure 12b show that both electron temperature and density increased with the voltage amplitude and frequency. Indeed, this rise in electron density at a higher applied voltage led to an increase in transported charges per voltage cycle and then to an enhancement in discharge efficiency [64].

Namely, when rising the external voltage amplitude from 15 kV to 30 kV, the electron temperature changed abruptly. Indeed, an important distortion of the electric field occurred above 15 kV due to the higher space charges emitted by the cathode. A significant rise in electron temperature results from this high gradient in the electric field distribution in the discharge gap [65]. After this abrupt increase, the electron temperature became less sensitive to changes afterward. As shown in Figure 12b, the electron temperature increased slightly with the external voltage frequency.

#### 4.5.2. Effect of Secondary Electron Emission Coefficients and Dielectric Constant of the Barrier Material

**Figure 13:**
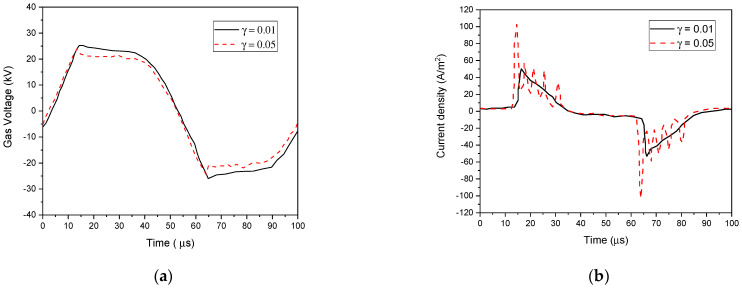
Calculated gas voltage waveform (**a**) and discharge current (**b**) at F = 10 kHz, V_app_ = 30 kV, and ε_r_ = 8.0 with different γ_i_ (0.01 and 0.05).

**Figure 14:**
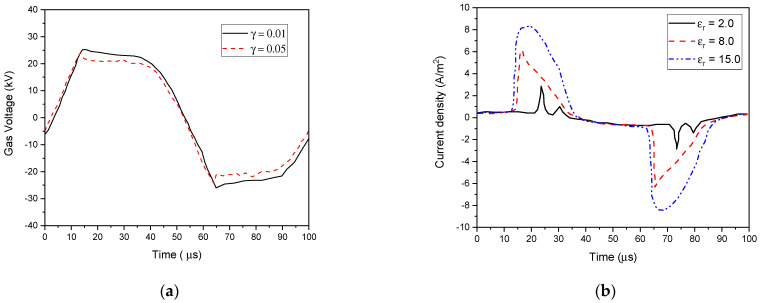
Calculated gas voltage waveform (**a**) and discharge current (**b**) at F = 10 kHz, V_app_ = 30 kV, and γ_i_ = 0.01 with different ε_r_ (2, 8, and 15).

The parameters of barrier material, such as the dielectric constant ε_r_ and the secondary electron emission coefficient γ_i_, can significantly alter the discharge characteristics. Their influence on the gas voltage and current properties is shown in Figure 13 and Figure 14 using He–O_2_ (1% O_2_) plasma gas. As illustrated in Figure 13b, the γi coefficient of the barrier material influenced the current waveforms in the discharge structure. Several peaks in the current waveform can be seen because of the rising γ_i_ coefficient from 0.01 to 0.05. It was found that the number of micro-discharges increased, which reduced their duration in response to a higher electric field [66].

The tendency shown in Figure 14 is the same as that observed when the external voltage amplitude was changed (see Figure 11a). Due to higher dielectric constants in the dielectric barriers, the voltage drop was smaller. This resulted in an enhanced plasma density at the same external voltage amplitude [67].

Consequently, plasma discharges can be operated at a low applied voltage and a high plasma density using higher values of γ_i_ and ε_r_ dielectric barrier materials. These results represent good guidelines to choose the suitable barrier material for each application.

#### 4.5.3. Influence of Oxygen Addition

**Figure 15:**
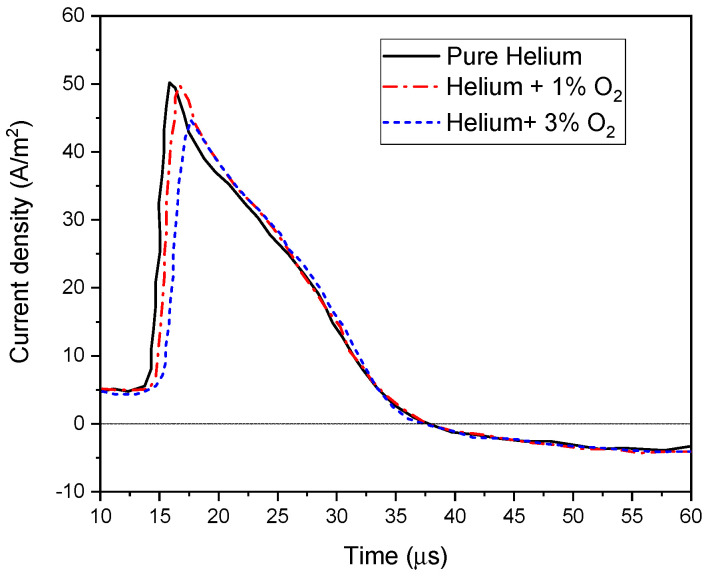
Current waveform distribution depending on oxygen addition at F = 10 kHz, V_app_ = 30 kV.

Figure 15 shows a slight decrease in the maximum current density and a widening current pulse as oxygen was added. On the other hand, by adding oxygen, electrons and metastable molecules were quenched, resulting in a decreased density for both species. Therefore, the inactivation efficiency dropped with the increase of oxygen addition, because the number of reactive species attending the polymer surface was reduced.

#### 4.5.4. Influence of the Discharge Gap

**Figure 16:**
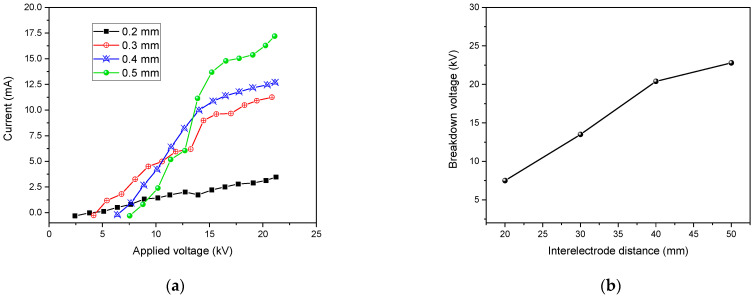
Variation of current of the DBD (**a**) and breakdown voltage as a function of the interelectrode distance (**b**).

In addition to the process gas, the operating distance also has a significant effect in the inactivation efficacy. In Figure 16, we positioned the polymeric samples under He–O_2_ (1% O_2_) plasma at different distances.

Figure 16a shows the characteristics of the current voltage DBD for several gap widths. A linear rise in the discharge current was illustrated with higher electrode gap distance and applied input voltage. For different applied voltages, the increase in the current was very small with a gap of 0.2 mm, but as the gap increased up to 0.2 mm, the current increase became linear. For a 0.3 mm electrode gap, the discharge was found to be more uniform, and a higher number of micro-discharges was observed [68].

Therefore, by increasing the distance, the inactivation efficiency dropped. Indeed, the longer path that the reactive species had to cover to attain the polymer surface and the small lifetimes for the oxygen atoms (in the order of milliseconds [69]) resulted in fewer reactive species attending the surface and possibly inactivating microbial growth. In addition, the area of the polymer strips covered by the plasma significantly decreased.

A linear relationship was found between electrode gap and breakdown voltage at atmospheric pressure, as illustrated in Figure 16b.

**Figure 17:**
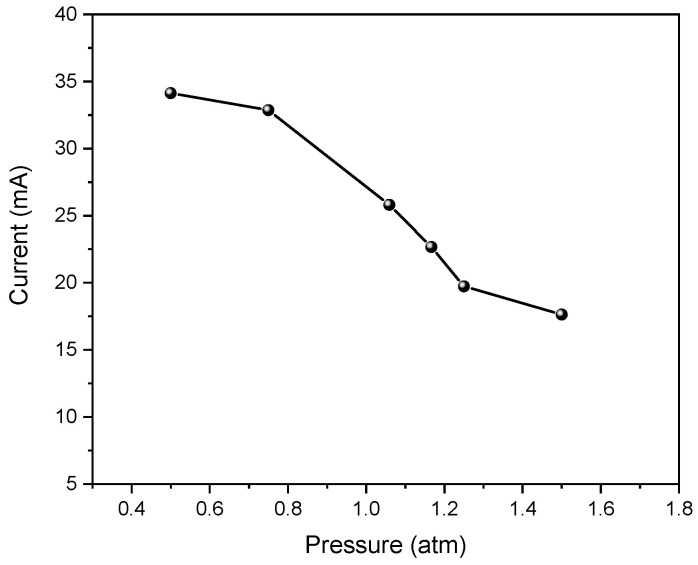
Discharge current variation at different pressures.

The breakdown voltage increased when the gas pressure increased, and the gap distance remained constant, according to Paschen’s law [70]. Meanwhile, as displayed in Figure 17, the discharge current gradually decreased with the rising pressure. This could be attributed to the lower generation rate of effective electrons during discharge, which indicated that surface sterilization was reduced under high pressure.

## 5. Conclusions

Throughout this research, a suitable non-thermal DBD discharge plasma at atmospheric pressure was developed for the bio-decontamination of polymer surfaces considered as heat-sensitive materials. This work was focused on the role of reactive species produced by helium and helium–oxygen plasma DBD in the inactivation of *Bacillus atrophaeus* spores. A 1D simulation model was developed using the COMSOL Multiphysics^®^ 5.4 package to examine the DBD characteristics and active species’ densities. The behavior of glow discharges including a single peak current in each half cycle was well reflected by the simulation results of the discharge current variation. A time-dependent analysis of electron temperature and density was carried out. The charged plasma species present in the discharge gap, significantly change the electric field and potential.

The species distribution between the plasma electrodes was obtained by numerically solving the transport of reactive species. Based on the substantial densities of (ROS) and RNS, it was revealed that they were liable for the surface treatment and plasma disinfection process. Bacterial death was mainly caused by membrane damage and DNA degradation induced by reactive oxygen species (ROS).

A glow discharge can be stable, with higher microbial purification efficiency, depending on the operational parameters, including external voltage amplitude and frequency, dielectric barrier thickness, and discharge gap width.

A higher applied voltage led to an increase in transported charges per voltage cycle and then to an enhancement in discharge efficiency. In addition, the electron temperature changed abruptly with the external voltage. On the other hand, it was possible to operate plasma discharges at a low applied voltage and a high plasma density using dielectric barrier materials with higher values of γ_i_ and ε_r_.

A linear relationship was found between breakdown voltage and electrode gap at atmospheric pressure. With increasing gas pressure, the breakdown voltage rises, and the current discharges declines at a constant gap width. Thus, a small gap width and the admixture of oxygen are needed for sufficient bio-decontamination. Plasma-based pollutant degradation devices could therefore benefit from these results.

## Data Availability

Data are contained within the article.
